# Translation and psychometric validation of the Chinese version of the meaningful and enjoyable activities scale for mild dementia

**DOI:** 10.3389/fpsyt.2023.1148838

**Published:** 2023-03-13

**Authors:** Hongyu Chen, Yuanyuan Wang, Minyi Zhang, Ning Wang, Xixi Hao, Zhihan Xue, Kui Fang, Yan Liu

**Affiliations:** ^1^Department of Neurosurgery, The First Hospital of China Medical University, Shenyang, China; ^2^Department of Nursing, Children's Hospital of Nanjing Medical University, Nanjing, China; ^3^Department of Nursing, The First Hospital of China Medical University, Shenyang, China

**Keywords:** people with mild dementia, meaningful activity, reliability, validity, Chinese

## Abstract

**Objectives:**

To translate 20-item Meaningful and Enjoyable Activities Scale into Chinese and evaluate its psychometric properties amongst Chinese with mild dementia.

**Methods:**

A cross-sectional study of 450 people with mild dementia recruited from a memory disorders clinic was conducted with the C-MEAS. Raw data were randomly divided into two parts for exploratory factor analysis and confirmatory factor analysis, to evaluate the construct validity. Content validity and reliability were tested by content validity index and Cronbach’s α coefficients, respectively.

**Results:**

Adaptation results showed that the Chinese version of the scale is adequate for linguistic and content validation. Confirmatory factor analysis indicated a significantly good fit for a three-factor model. Cronbach’s alpha coefficient was 0.84 for the overall scale.

**Conclusion:**

The C-MEAS for people with mild dementia is a reliable and valid instrument with satisfactory psychometric properties. Future studies should recruit a more representative sample of people with mild dementia in China to verify the applicability of the scale.

## Introduction

Dementia is a syndrome characterized by a chronic or progressive decline in cognitive function that worsens faster than normal aging expectations, with adverse physical and psychological effects on the patient (1). People with dementia may gradually lose the ability and opportunity to engage in various activities due to decreased cognitive function, difficulty expressing their needs (2), and a lack of care resources (3). A UK study shows that people with dementia spend less than 12 min a day engaging in meaningful activities (4). There is still insufficient attention to meaningful activities in existing dementia care plans (5). Furthermore, the current optional activities offered in the community or nursing home setting are insufficient to meet the needs for stimulation and interest among people with dementia (6). According to the developmental psychology theory of aging, providing meaningful activities for people with dementia is not only to bring more pleasure but also to meet their psychological needs (7).

Meaningful and pleasurable activities are those that provide emotional, creative, intellectual, and spiritual stimulation. These activities can include physical, social, and leisure activities that are tailored to individual needs and interests (8). Although engaging in meaningful activities is an important part of person-centered nursing practice (9); however, this need is often not given the attention it deserves (10).

Research shows that meeting the needs of people with dementia to engage in meaningful activities can improve quality of life (11), reduce behavioral and psychological symptoms and functional dependence (12–14), maintain identity (15), increase happiness, and interest (16), increase caregiver well-being, and improve relationships with caregivers (17, 18). Conversely, a lack of access to meaningful activities, or an inability to participate effectively, has been associated with worsening behavioral and psychological symptoms, such as agitation, depression, apathy, denial of care, and a lower quality of life (3, 17). Activities that are too simple can make people with dementia feel bored and reluctant to participate, and activities that are too complex can lead to frustration. Therefore, assessing the meaningful and enjoyable activities of people with dementia and exploring boundaries are crucial issues as they allow healthcare workers and family carers to develop activity management strategies and prevent negative outcomes.

People with mild dementia are better suited to a wide range of activities than people with moderate to severe cognitive impairment. This may be related to factors such as more social interaction and less cognitive deterioration (19). The Meaningful and Enjoyable Activities Scale (MEAS) was the first validated tool used to measure meaningful activity for people with mild dementia (20). Guided by the psychological theory of aging, it combines expert opinion, carer feedback, and patients’ daily experiences, and has been shown to have good reliability and validity. Due to the lack of validated scales available in China, this study translated the MEAS into simplified Chinese and tested its reliability and validity.

## Methods

### Ethical considerations

The study was approved by the Research Ethics Committee of the First Hospital of China Medical University (No.2021434). Before participating, all people with dementia and their carers were given a detailed introduction to the research background and purpose. Participation was voluntary and could be withdrawn at any time. The confidentiality of participants is ensured by anonymity. Sign the informed consent form to express consent to participate. The data collected is only used for this study.

### Participants

We recruited people with dementia and their family carers through the memory disorders clinic of The First Hospital of China Medical University. Participants were recruited if they: (a) had a diagnosis of mild dementia of any type (Mini Mental State Examination Score—[MMSE] ≥ 21) (21); (b) lived in the community; and (c) had a family carer who was able to take part and act as an informant. Questionnaires including personal information forms, the Chinese version of the MEAS, and informed consent were provided to caregivers. Among the sociodemographic characteristics of the participants are age, gender, type of dementia, and MMSE score. The sample size estimates in the factor analysis were based on a rule of thumb with a minimum of 10 respondents per item (22). The minimum sample size required for MEAS is 200 participants since there are 20 items. A total of 465 family carers were invited to participate, and 450 family carers completed the entire survey, which showed adequate samples for exploratory factor analysis (EFA) and confirmatory factor analysis (CFA) (23). Reasons for refusing participation included lack of time to participate and lack of interest.

### Instruments

The MEAS developed by Vasiliki Orgeta et al. (20) is a proxy-reporting scale that includes leisure-time physical activity, social engagement, and mentally stimulating activities as key dimensions. It consists of 20 items, with options using a 5-point Likert scale, ranging from 0 (never) to 4 (almost daily), and the total score ranges from 0 to 80. Higher scores indicate higher levels of meaningful activity. In the UK, the Cronbach’s alpha was 0.79 for the total, indicating good psychometric properties.

### Psychometric testing procedures

Using the original version of MEAS was authorized by the author *via* email. The commissioning process of the MEAS scale was determined according to the cross-cultural commissioning scale guidelines recommended by the American Society of Orthopaedic Surgeons Evidence-Based Medicine Committee (AAOS) (24).

To translate the scale into Chinese, two bilingual translators completed a forward translation of the original version. The research team then evaluated the two translated versions and produced an integrated version after discussion. Next, two additional translators who had never seen the original scale completed a back-translation of the integrated version into English. The two back-translated versions were compared with the original scale to assess semantic equivalence, resulting in a preliminary version of C-MEAS. The bilingual translator team consisted of two chief physicians of neurology and two English teachers who are knowledgeable about colloquialisms and slang.

We designed the expert consultation letter, which required experts to evaluate the relevance and clarity of each item on a 4-point Likert (1 being irrelevant/clear to 4 being highly relevant/clear). Experts were asked to provide alternative expressions for items rated 1 or 2. In total, seven experts were invited and participated in the study, including areas of neurology, geriatrics, and dementia care. Some items have been revised based on expert opinions and the cultural background of our country. ‘Going out for a coffee or a meal or other social event’ and ‘Doing crosswords or puzzle’ was revised to ‘Going out for a tea or a meal or other social even’ and ‘Playing Mah-Jong or chess’. Content validity was estimated through content validity indexes at the item level (I-CVI) (reference range ≥ 0.78) and the scale level (S-CVI/ave) (reference range ≥ 0.90) (25).

A pilot experiment was conducted with 10 carers of people with mild dementia. They were asked through cognitive interviews whether they understood the items to determine whether the content of the C-MEAS was clear and understandable. Based on feedback suggestions, ‘Light exercise (i.e., walking, yoga, light housekeeping)’ was revised to ‘Light exercise (i.e., walking, Tai Chi, Square dance, light housekeeping)’. These carers were not included in the study sample. A tentative version of the C-MEAS scale with 20 items was formed, and it was sent to 10 healthcare providers (including nursing assistants, nurses, and doctors) working in nursing homes and geriatrics for face validity assessment.

### Data analysis instruments

The collected data was analyzed using IBM SPSS STATISTICS 25.0 and AMOS 24.0. Descriptive statistics were used to assess the participants’ demographic characteristics. The discrimination ability of the C-MEAS scale was assessed by item-total scale correlation, and a correlation coefficient below 0.3 was suitable for deleting items (26). Internal consistency reliability was measured using Cronbach’s alpha coefficients, and alpha values >0.8 were considered ideal (27).

Using the EFA and CFA, construct validity was assessed. Raw data were divided into two groups at random. Part 1 (*N* = 220) examined the factorial structure of C-MEAS, and part 2 (*N* = 230) confirmed EFA results. Prior to EFA, the sampling adequacy was tested using the Kaiser-Meyer-Olkin (KMO) and Bartlett’s spherical test. Factors with a factor load >0.40 and an eigenvalue >1.0 were extracted. To test the goodness of fit, this study used chi-square/degrees of freedom (*X*(2)*/*df, cut-off <3), root mean square error of approximation (RMSEA, cut-off <0.08), comparative fit index (CFI, cut-off ≥0.95), goodness-of-fit index (GFI, cut-off ≥0.85) and incremental fit index (IFI, cut-off ≥0.90) (28).

## Results

A total of 450 carers completed the survey, and Table 1 shows the characteristics of the participants.

**Table 1:** Demographics of people with dementia and family carers (*N* = 450).

	Mean (SD) or *N* (%)
People with dementia	
*N* = 450	
Age (years)
50–59	36 (8)
60–69	215 (47.8)
70–79	153 (34)
≥80	46 (10.2)
Sex
Female	237 (52.7)
Dementia type
Alzheimer’s disease	356 (79.1)
Other	94 (20.9)
MMSE	23.4 (1.7)
Carers
*N* = 450	
Sex	
Female	263 (58.4)
Age (years)
50–59	45 (10)
60–69	231 (51.3)
70–79	139 (30.9)
≥80	35 (7.8)
Relationship to participant
Spouse/partner	354 (78.7)
Child/Child in law	81 (18)
Other	15 (3.3)

### Content and face validity

I-CVI was calculated by dividing the number of experts giving ratings of 3 or 4 by the total number of experts. S-CVI was calculated by taking the average of I-CVIs. Analyses showed that I-CVI values of the C-MEAS ranged from 0.86 to 1, and the S-CVI was 0.96, indicating acceptable content validity. Healthcare providers were also asked for their feedback on the clarity of the content of the C-MEAS. They stated that the questions were easy to understand and answer.

### Construct validity

Item-total correlation coefficients of the C-MEAS scale ranged from 0.360 to 0.661, which were all statistically significant. If any item was deleted, the alpha value of the whole scale was reduced, indicating all projects are suitable for inclusion in EFA. The KMO coefficient was 0.813, and Bartlett’s test was significant (*p* < 0.001), which supported conducting EFA (29).

EFA suggested a three-factor solution, which explained 57.401% of the total variance. The factor loads of the three-factor model of the C-MEAS ranged between 0.479 and 0.840. Table 2 details which items are loaded on the three-factor.

**Table 2:** Item-total correlation, reliability coefficients and factor loads of the C-MEAS (*N* = 220).

Factor name	Item	Item-total correlation	Factor loads	Cronbach’s alpha if item deleted	Cronbach’s alpha
Leisure physical activities					0.773
	Going for a walk	0.364^**^	0.490	0.840	
	Light housekeeping	0.404^**^	0.826	0.837	
	Light exercise	0.433^**^	0.479	0.837	
	Relaxation or stretching exercises	0.380^**^	0.738	0.838	
	Taking care of plants or gardening	0.374^**^	0.782	0.839	
	Engaging in self-care	0.411^**^	0.734	0.837	
Mental activities					0.910
	Reading magazines/books/newspapers or other	0.579^**^	0.736	0.830	
	Keeping up with current affairs	0.612^**^	0.806	0.828	
	Listening to music	0.525^**^	0.797	0.833	
	Watching films/movies or television programs/documentaries	0.595^**^	0.827	0.829	
	Playing Mah-Jong or chess	0.638^**^	0.840	0.826	
	Drawing, painting, arts and crafts or other engagement with the arts	0.661^**^	0.774	0.825	
	Going to a place of worship	0.613^**^	0.707	0.828	
	Watching wildlife or being close to nature	0.623^**^	0.770	0.827	
Social activities					0.847
	Engaging in voluntary, political or other social/community activities	0.360^**^	0.727	0.839	
	Phoning family and/or friends	0.499^**^	0.752	0.833	
	Being with children or grandchildren	0.411^**^	0.796	0.838	
	Having friends over or visiting friends	0.556^**^	0.814	0.831	
	Going to the local shops	0.399^**^	0.657	0.838	
	Going out for a tea or a meal or other social event	0.449^**^	0.711	0.835	
Total					0.840

**p<0.01.

**Figure 1:**
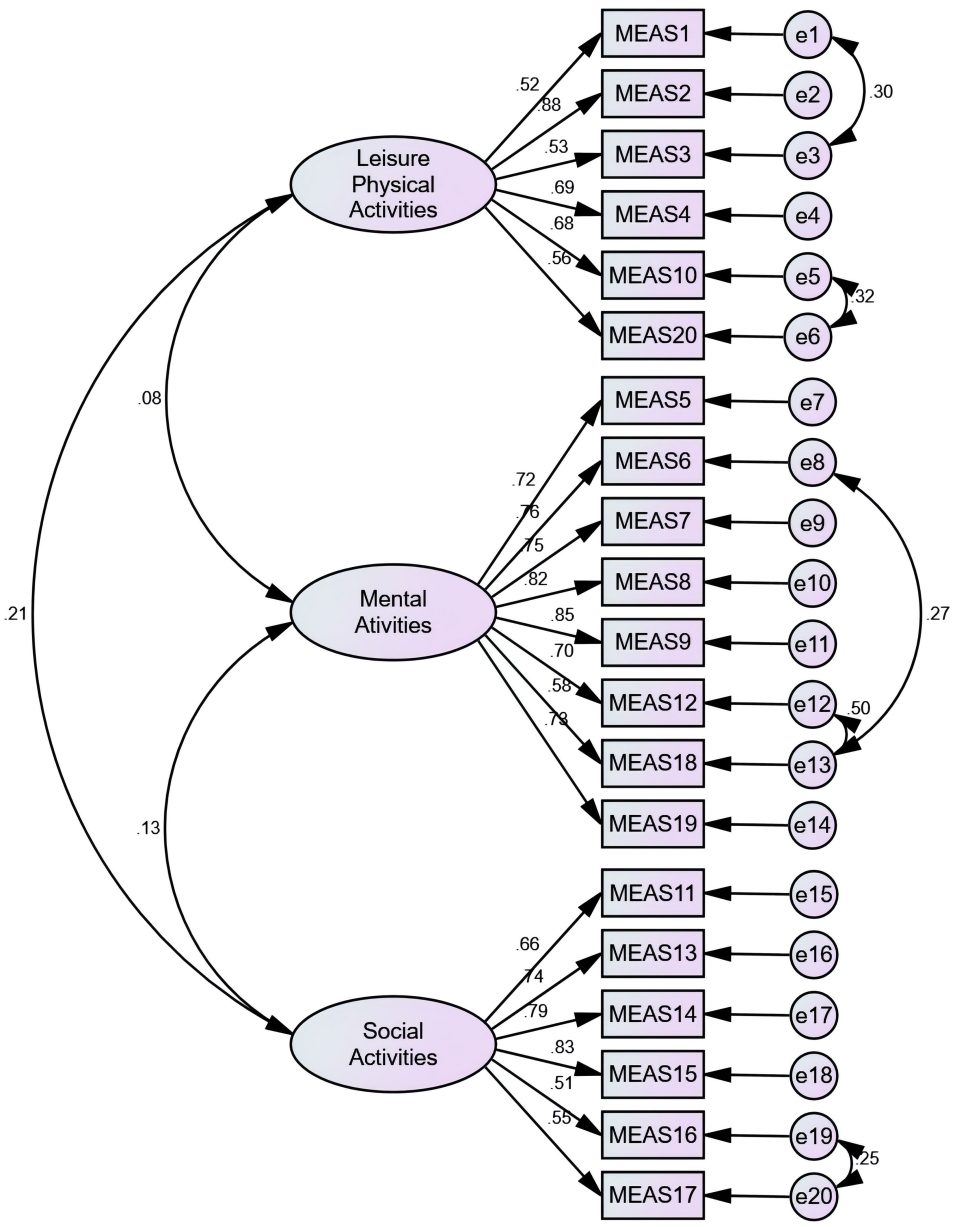
CFA of the modified three-factor model of the C-MEAS (*N* = 230).

The three-factor model was examined with part 2 data (*N* = 230) using CFA. The primary fitting index wasn’t meet the fitting standard. According to the modification indices (MI), the initial model was revised five times in the following order: e1 and e3, e5 and e6, e8 and e13, e12 and e13, e19 and e20, respectively. Based on goodness-of-fit statistics, the adjusted three-factor model was acceptable (Figure 1). The C-MEAS model fit indices for the primary and secondary models are shown in Table 3.

**Table 3:** Fit indices of the models (*N* = 230).

Fit indices	Good fit	Acceptable fit	Three-factor model	Adjusted three-factor model
*χ*^2^/df	1 < *χ*^2^/df < 3	3 < *χ*^2^/*df* < 5	2.927	2.307
RMSEA	0 ≤ RMSEA ≤0.05	0.05 < RMSEA <0.08	0.092	0.076
CFI	0.97 ≤ CFI ≤1	CFI ≥ 0.90	0.855	0.905
GFI	0.90 ≤ GFI ≤1	0.85 ≤ GFI <0.90	0.833	0.870
IFI	0.95 ≤ IFI ≤1	IFI ≥ 0.90	0.857	0.906

*χ*^2^/df, chi-square/degree of freedom; RMSEA, root mean square error of approximation; CFI, comparative fit index; GFI, goodness of fit index; IFI, incremental fit index.

## Discussion

Building meaningful and enjoyable activities is an integral part of dementia care, as it promotes the quality of life for people with dementia (19). The MEAS scale was translated into simplified Chinese and measured psychometrically among 450 Chinese with mild dementia. The C-MEAS is a feasible, appropriate quantitative tool for people with mild dementia, as all participants reported that the scale items were easy to comprehend.

In general, this study’s recruited sample of people with mild dementia showed satisfactory reliability and validity for the C-MEAS. All item-total correlation coefficients were above 0.3, which displayed good discriminating abilities. Besides, a Cronbach’s coefficient of 0.840 was found for the total scale, slightly better than the original version, which was 0.79 (20). Different cultures and habits of activity may have influenced the research results. Among Chinese people with mild dementia, the C-MEAS showed acceptable internal consistency reliability with Cronbach alpha values between 0.773 and 0.910.

EFA results grouped 20 items under three factors, which explained 57.401% of the variance. The factor loads for items were 0.40 or higher, which is considered ideal (30). It was found that some residuals were correlated, making the initial model less ideal. In order to adjust the primary model, correlations between some items were established based on modification indices. As a result of making appropriate adjustments and corrections to the original model, the data fit the adjusted three-factor model well.

With its positive psychometric properties, the C-MEAS can be used to evaluate the meaningful and enjoyable activities of people with mild dementia accurately and reliably. Therefore, future research can use the C-MEAS to support the construct of meaningful activity in people with mild dementia.

There are some limitations in the present study. We recruited individuals with mild dementia who are typically active, so our findings may not be generalizable to those with impaired physical mobility. In addition, we have not yet compared our scale to other existing measures to assess concurrent validity, as there is no commonly accepted gold standard for evaluating meaningful activities worldwide. Furthermore, we did not investigate the factors that influence meaningful activity engagement among people with mild dementia, which is a crucial area for future research.

## Conclusion

The English version of MEAS has been successfully introduced into China after translation and cross-cultural debugging, and its psychometric characteristics have also been verified in people with mild dementia. In addition, factor analysis shows that C-MEAS has appropriate reliability and validity. Under the background of aging, as the country with the largest number of people with dementia in the world, it provides an effective assessment tool for improving the activity level of patients with mild dementia and provides a basis and premise for subsequent research on targeted intervention to delay the progression of cognitive impairment.

## Data availability statement

The raw data supporting the conclusions of this article will be made available by the authors, without undue reservation.

## Ethics statement

The research follows the basic principles of clinical ethics. Approved by the Ethics Committee of the First Hospital of China Medical University (approval number: [2021]434), Written informed consent was obtained from all people with dementia and their carers.

## Author contributions

HC and YL conceptualized and designed the project. HC, YW, NW, and YL acquired and managed the data. XH, ZX, and MZ performed statistical analysis and data analysis. HC and KF drafted the manuscript. YL and NW revised the manuscript. All authors contributed to the article and approved the submitted version.

## Funding

This study was supported by the Natural Science Project of The Educational Department of Liaoning Province (FWZR2020004).

## Conflict of interest

The authors declare that the research was conducted in the absence of any commercial or financial relationships that could be construed as a potential conflict of interest.

## Publisher’s note

All claims expressed in this article are solely those of the authors and do not necessarily represent those of their affiliated organizations, or those of the publisher, the editors and the reviewers. Any product that may be evaluated in this article, or claim that may be made by its manufacturer, is not guaranteed or endorsed by the publisher.
